# FINDINGS FROM ASSESSING AGE-FRIENDLY BARRIERS, STRENGTHS, AND AREAS OF OPPORTUNITY AT WESTERN OREGON UNIVERSITY

**DOI:** 10.1093/geroni/igae098.1682

**Published:** 2024-12-31

**Authors:** Melissa Cannon

**Affiliations:** Western Oregon University, Monmouth, Oregon, United States

## Abstract

This presentation showcases the work that has been done at Western Oregon University (WOU) as an Age-Friendly University (AFU) member related to assessment and identifying action items for increasing age-friendliness and lifelong learning opportunities. First, attendees will learn about a multi-method case study that explored barriers, facilitators, and opportunities for WOU to engage older community members and advance age-friendliness in preparation for joining the AFU network in 2019. This case study involved survey data collection and participatory action research with older adults, revealing opportunities for the university to implement the AFU principles through efforts such as strengthening the senior center partnership, developing a lifelong learning center, and removing accessibility barriers. Next, this presentation will delve into findings from WOU’s “Age-Friendly University Campus Report” (Silverstein et al., 2021), which helped WOU identify strengths, weaknesses and areas of opportunity related to lifelong learning. For example, the report found a lack of resources and information for campus community members who identify as informal or unpaid family caregivers at WOU, which can particularly impact non-traditional students with family caregiving responsibilities. Findings will also be shared from the latest AFU-related study at WOU which explored caregiving issues for campus community members (e.g., physical demands, time demands, financial stress, social and emotional health) using a campus-wide online survey and mixed-method analysis by a research team. After providing this overview, next steps will be explored as to how WOU and similar universities may take action to implement changes that enhance opportunities for lifelong learning on campus.

